# A Netnographic Study of Consumer Value in Slow Travel

**DOI:** 10.1007/978-3-030-65785-7_15

**Published:** 2020-11-28

**Authors:** Veera Riikonen, Juho Pesonen, Johanna Heinonen

**Affiliations:** 1 Department for Informatics, Technical University of Munich, Garching bei München, Bayern Germany; 2 Smart Tourism Education Platform (STEP) College of Hotel and Tourism Management, Kyung Hee University, Seoul, Korea (Republic of); 3 Department of Tourism and Service Management, MODUL University Vienna, Vienna, Wien Austria; Business School, Centre for Tourism Studies, University of Eastern Finland, Yliopistonkatu 2, 80100 Joensuu, Finland

**Keywords:** Customer value, Tourist experience, Netnography, Travelling by land, Slow tourism, Facebook groups, Sustainable tourism

## Abstract

Travelling by land is a phenomenon that utilizes different surface transport modes, such as trains, buses, bicycles etc. The slow travel contributes also to the concerns about ecological footprint and climate change derived from air travel. Slow travel aims to encourage individuals to travel to their destinations more slowly, stay for a longer period in the chosen destination, and travel less. For slow travellers, travelling to the destination is a significant part of the travel experience. The qualitative research aimed to understand the phenomena of travelling by land and the tourist experience holistically using a netnographic approach. The data was collected from the Finnish Facebook -group, Maata pitkin matkustavat. (Those who travel by land) in January 2020. The data consisted of 185 posts and their comments. The goal of the data analysis was to understand the role of consumer value in the slow travel experience. The research findings show the importance of minimizing travel time and the costs of travelling by land. Also, leisure time, and “having fun” are valued in travelling by land experience. Thus, self-oriented, active value components, Efficiency, and Play, were most applicable in the collected data set. These findings help us to understand slow travel as a tourism experience better and provide important insights into the requirements to develop consumer-centric slow travel for sustainable development in the future.

## Introduction

### Background

The tourism industry has a sustainability problem. Before the COVID-19 pandemic, the transport-related emissions from tourism represented 5% of all man-made emissions [31]. Especially air transportation often required by tourism has been seen as one of the main sources of CO2 emissions in the industry. Most studies examining the environmental impact of tourism often neglect the effects of travelling to a destination (and back), and mostly just focus on tourism’s effects at the holiday destination. As Böhler et al. [3] state that although car travel still dominates the holiday mobility, increasing global tourism there has been a significant demand for growing holiday air travel [3]. Thus, to reduce CO2 emissions alternative forms of transportation are needed and understanding their appeal for consumers is a major factor in succeeding to increase alternative transport method use in tourism.

The concept of slow travel arises in this context as a response to the concerns about the ecological footprints and climate change derived from air travel. Slow travel aims to reshape the notion of sustainable destinations and conceptualize slow travel as an alternative to travel by car or flying. Slow travel encourages individuals to travel to their destinations by land favoring public transport modes, stay longer in the chosen destination, and travel less [20]. Slow travel could provide solutions for creating a thriving tourism industry with less greenhouse gas emissions, at least in some destinations.

This paper aims to study consumer value in slow travel through a netnographic study of a social media community focused on travelling by land. The research question we aim to answer is *How tourists experience the consumer value of travelling by land?* This research question allows us to explore what tourists value in their slow travel experience [25]. Finland has set an objective for being one of the top countries is sustainable tourism by 2025 [35], which requires attitude change from customers as well. Slow travel is a relatively new concept, there is little research about the topic, which is one of the motivations for this study. Thus, understanding consumer motives and value that they experience from slow travel can contribute to wider adoption and use of sustainable transportation in tourism.

## Theoretical Framework

### Travelling by Land as a Tourist Experience

To evaluate the tourist experiences and the value building in travelling by land, one must understand the concept of tourist experience and consumer value. To consume tourism is to consume experiences [29]. Experiences, however, are not similar to all tourist, even with the specific context and places. Tourist experiences are unique to individual tourist. There are as many tourist experiences as there are tourists. Tourist experiences are individualistic and they engage emotions, which is essential in creating a memory. [32] The extraordinary experiences are characterized by emotional intensity [1].

In this study, the tourism experience does not only include on-site experiences. Also, the pre-trip phase is part of the experience. Both plans (before) and memories (after) are essential parts of the tourist experience [26]. Travelling to and from the destination are viewed as integral parts of the experience, and not separate experiences or inseparable “costs” of the experience. Especially in the case of travelling by land as a tourist experience, being on the road and travelling as a means to dislocate are as important factors than the destination itself [26].

In this case, we adopt the consumer value framework to improve our understanding of travelling by land. Sánchez-Fernández & Iniesta-Bonillo [28] argue that “value” is a simple trade-off between benefit and sacrifice. However, Williams and Soutar [34] acknowledge that in services, due to their nature of being intangible, heterogeneous and complex, the trade-off model is too simplistic.

According to Holbrook [10,11], a consumption experience may create value for the customer. Holbrook has shown a long and consistent interest in the topic of value [6]. According to Holbrook, consumer value is defined as an interactive, relativistic, preference experience [10], emphasizing the interaction between the product and user from which value is derived. This definition assumes that consumers purchase products and services to achieve value-related goals or to obtain their benefits [12]. Typology of value is divided into eight separate categories of consumer value: efficiency, excellence (quality), play, aesthetics, status, esteem, ethics and spirituality. Distinct categories are based on the three-dimensional paradigm [6] consisting of extrinsic and intrinsic value (utilitarian vs. Hedonistic), self-oriented and other-oriented value, (when in a consuming act includes a social dimension) and active and reactive value (active or passive control of the customer on the object) [6,10]. Both consumers value appreciations and priorities have a strong influence on the way consumer ultimately perceives an experience [12].

Evaluating the customer value in this study is done by using Holbrook’s [10] framework of consumer value to identify the different value components of the experience in travelling by land. However, Holbrook’s theoretical proposal does not consider negative dimensions of value, which is criticized by his co-authors and admitted by himself [10,12].

### Slow Travel as a Tourist Experience

Travelling by land is travelling without flying and thus connected to slow travel. Slow travel can be defined as a conceptual framework that focuses on people who travel slowly overland, stay longer and travel less [4,20]. Slow tourism emerges from concern for environmental sustainability and traveller’s personal and social well-being. Interest in slow tourism lies in the transport to and from destinations that could reduce environmental pollution by using low carbon emission vehicles. Therefore, slow tourism is frequently tied to sustainable tourism [24]. However, the phenomenon needs to be understood also in a broader socio-cultural context of the slow movement, [25] situated in the context of contemporary consumption society [27].

As well as tourist experiences in general, slow travel experiences include the phases before, during and after the trip. Dickinson and Lumsdon [4] emphasize that definitions of slow travel should focus on both transportations for the sake of protecting the environment, as well as participation in the slower forms of travel, for example; exploring local history, culture and people [4]. Compared to mass tourism which extensively promotes the use of transportation with no attention to the environment, and where vacations are experienced in a standardized manner, slow travel enables people to travel at their own pace, being able to avoid fixed schedules if preferred. Slow travellers can be considered as independent, tough, resilient, and eager for new experiences [4,27].

The mode of transport is a meaningful part of the whole tourist experience and it is equally important as is the stay in the destination itself [20,24,27]. Lin [19] found in her research, that the tourism experience drives slow travellers to be more engaged with sustainable tourism. Slow travel experiences structure tourists’ time and enable tourists to engage locations and locals deeply and develop quality leisure moments. Accessibility, ease, pleasure, safety, and informative guides at the destinations, are factors increasing the willingness of industrial tourists to try slow tourism [19].

Slow travel is related to less travel intensive tourism [27] while slow tourism goes beyond mobility and the mode of transport. Slow tourists expect to see more than a gaze of their destination and instead, they are more likely to have more immersive experiences. Emphasis is on quality over quantity [20].

The empirical research regarding slow tourism is still lacking. Interviews have been used to develop frameworks that help us to understand the phenomenon better [21]. Oh and his colleagues [25] used interviews, focus groups, and a survey with 1068 respondents to study the motivations and goals of slow tourism. They found that tourists can set slow tourism as a goal and the maximize attainment of some superordinate goals through optimal choices related to travel. Tourists constantly compare choices in the slow-fast continuum to find maximal goal attainment. Lin [19] identified that the quality of the transportation mode and the tourism experience are the most powerful determinants of slow tourism intentions among industrial tourists in Taiwan. Sales Oliveira [27] analysed the discourse of slow travellers using their travel blogs. She found that subjective perceptions and representations of slowness are key elements in slow tourism experience. However, with slow travel discourse, the juxtaposition with instantaneous time is nonetheless present.

## Research

### Target Group and Data Collection

Understanding why people choose slow travel is needed to develop slow, sustainable tourism. Social media has become an important communication venue for people to express themselves. Thus, this study focuses on a Finnish Facebook group called “Maata pitkin matkustavat” (Those who travel by land) [22]. The description of the group is “We want to travel environmentally friendly, without planes!”.

The group’s slogan supports Visit Finland’s goals of sustainable tourism in Finland [35] and this kind of group provides qualitative discussion data of the topic, without being affected by the research at hand. The discussion comes naturally from the participants in the group and describes the motivations and values of people interested in the topic of slow travel.

The qualitative data from discussions aim for a holistic understanding of the issues studied. The research is also conducted in an abductive manner that moves from everyday descriptions and meanings to categories and concepts, which will create the basis for understanding the phenomena described [5].

The research starts with the data, which is already existing and created by the members of the Facebook group. Maata pitkin matkustavat - Facebook group is founded in 29.6.2015. When this study started, the group had 16 189 members (26.9.2019). During the data analysis (4.9.2020) the group had grown to 22 315 members.

Data for the research was collected from the conversations and posts in the group, made in January 2020. The data collected consisted of 185 posts, including the comments. The research started from the assumption the members in the group have already decided on the travel mode, to travel by land and avoiding flights as they were members of the group. The discourses and themes discussed in the Facebook group will answer the research question defined and help to understand travelling by land as a phenomenon. The data was saturated as adding new posts to the data set did not bring any new insights into the results.

### Methodology

Netnography was chosen as the research approach as it allows a deep understanding of the culture of the social media community. As in ethnography, netnography adapts research techniques to study online consumer-based communities [33]. The cornerstone of the research process is participant observation [2,18,23]. Netnographic research does not evaluate the interactions between the participants [33]. It is interesting in what kind of topics and discourses received comments and likes in the group, and to which kinds of topics the discussion in the community is focused on.

Netnography is an established approach for qualitative research [15]. The name draws from two terms, “internet” and “etnography” [14]. The approach has several similarities to ethnography, from which it has been adapted. *“It is a qualitative, interpretive research methodology that adapts traditional ethnographic techniques to the study of social media“* [16]. Netnographers role is limited to analysing the existing data (online material) [30].

Netnography is a flexible method, allowing scholars to explore and explain rich and diverse cultural worlds [15]. Netnographic field sites are diverse, [15] yet the focus in this research is to only one site, a Facebook group which can be defined as a social networking site. Online data can provide insights into a naturally occurring community [14] and makes it easier to reach the population which might otherwise be difficult to reach for consumer research [24,36]. Originally netnography is developed as a response to customers increased internet use [13]. It helps to understand the consumption-related aspects of customer’s lives online.

In this research netnography was the used approach and analysis of qualitative online data, i.e. Facebook posts, was done by using thematic analysis as well as content analysis. The thematic analysis involves coding and categorizing the data for emerging patterns and themes [7]. Once the data was collected, each of the posts and their comments were carefully read and examined. The posts were analysed in chronological order, from older to the latest. Authors wrote notes to each of the posts and categorized the posts, based on the value component the posts represented, and the main topics from the posts. In the analysis, Holbrook’s [10] framework of consumer value was used to categorize the posts under different value components. In the data, some of the posts represented not only one value component [10], but several of them.

### Findings

Members in the group were active and there was lots of discussion around the topic of travelling by land. It highlights the fact that the phenomena – travelling by land – is current, and there is lots of interest around it. Since people are eager to find out more and search for practical tips, it can also be noted, that the way of travelling (by land, avoiding flights) is a relatively new phenomenon and alternative way to travel for many. Many users need peer-support in organizing the trips without flying. This also highlights that travelling by land is not (yet) made as easy as it is to travel by plane but requires relatively more planning and effort before the actual travel begins. Some of the members are also ready to travel long distances by land. In the data, some of the longest journeys and distant destinations members are planning to visit/or had visited were Japan, Vladivostok, Iceland, and the USA. The conclusion of the increasing interest around the topic can also be drawn from the increased member number in the group, during the research process.

The writers of most of the 185 posts adopted a detailed mixed text and image style presentation. There was a notable amount of posts (70) including links to different websites, most of them news articles, blog posts related to travelling (by land) or YouTube videos related to the groups' common interest. Members invariably used their photographs in the posts. Since members post pictures and stories from different destinations all this planning of travelling by land has led to action as well. Pictures of train timetables and maps were posted as well, to ask for more specific information from other members or other experiences of particular timetables or routes.

Generally, members of the group are eager to help one another and tell about their own experiences in a very detailed manner. The discussions are mostly positive and encouraging. Since there were a lot of posts asking for tips and recommendations for different routes or places to visit while travelling, members seem to trust the peer-support of other members in the group. The comments are supportive, and some members take their time to compare the routes for each other and comment with different alternatives. There is some discussion about the destinations too, and what to see on the way. In some posts, there were even recommendations for the music for the road, to get into the mood of the destination. Not only are the discussions encouraging, but members also seem to be keen to know real facts and are looking for scientific information about the issues discussed in the group. If it is about the emissions on different travel modes, some of the members often compare the different result from different sources. They also do not trust completely to different surveys results posted to the groups but try to aim to understand possible flaws and errors in results. Sometimes also the sources for some controversial statements are asked from other members. Some members can be seen as agents of change, driving the agenda of the group.

By far the dominant topic in the discussion is travelling by train. Most likely it is the most convenient way of travel and there is a vast rail network within Europe, where most of the members primarily travel. (Maata pitkin matkustavat 2020) There is also a lot of discussion regarding the Interrail travel. It needs to be noted, however, that travelling by land includes also other transport modes, such as buses, private car, ferries, ships, and bicycles. All the mentioned were presented in the data as well, but in the lesser extension than the discussion about the trains.

The discussions in the group form a massive “data bank.” From the earlier posts, members can search for topics they are interested in or need more information. By reading the older posts, member do not necessarily need to make a post of their own, but they can use the already existing information, shared experiences, and practical tips, for example in planning their holidays. Since there are many posts made in a single day, in some of the posts chosen for the analysis, the comments suggested the member who made the original post to “search” for the group and the previous discussions about the topic. There is one post telling;

“*Our family has decided to travel to Malaga by land this time and hopefully in the future as well. The group has been a great help in planning the trip and choosing the places to visit. Group has been helpful in the problematic situations. Thank you for the inspiration”.*

There was no specific information asked in the post related to the trip, but to thank about the group in general. From this example, it can be interpreted that since there is already a lot of discussion regarding planning the routes all over Europe in the group, members can use the existing information when planning their trips. In some posts, members also commented with website links to other pages where to find more information, their travel blogs, Instagram pages, or other Facebook-pages related to travelling by land.

Not only are the older posts beneficial, but in the data set, there were new posts made which could be interesting to the group to know. These include newspaper articles about the new train routes under consideration, maps showing all the night trains around the world, mentions about current a radio or TV programs and so forth. The post types varied from blog posts or YouTube Channels to news articles as well as comments and texts written by a member. All the posts, which only included a link to another website, or only a picture, with no explanation or writing from the member who did the original post, were excluded from the research. Research findings also could mirror several value dimensions of travelling by land experience. Some of the posts were categorised under one value component, and some included even four different value components.

Table 1 shows how often different value components were applicable in the posts as well as explains how they were presented in the data. Play and Efficiency were the most represented and spirituality the least. Some of the posts were categorized under several value components. This was noted by Holbrook [10] himself too, that any consumption experience can entail many or even all the different types of consumer value, which are identified in the typology (Holbrook 1999, 186).

There were also negative issues discussed in the dataset, which Holbrook’s [10] typology do not take into account. These concerned usually the extra time that travelling by land takes in comparison to flying to the destination. Also, the different route options caused insecurity and indecisiveness. Other negative posts discussed e.g. strikes, maintenance works, visas and prices.

**Table 1.** Value components represented in the data set and their realization in travelling by land experience

Dimension	Number of representations	Realization
Play	100	Having fun while travelling, travelling for a holiday, exploring new places and cities on the way, visiting museums, amusement parks, beaches, natural parks, enjoying restaurants and culture
Efficiency	92	Time and cost variants; being able to travel as fast as possible from A to B, choosing the most inexpensive tickets for train/bus while travelling, saving money by travelling by land (instead of flying)
Status/Esteem	32/37	Successfully making a trip travelling by land, having a blog, Instagram page, or YouTube channel about travelling by land (the possibility to influence others while making one’s own consumption patterns visible in social media) Reputation traveller receives from others by choosing the surface travel modes
Ethics	25	Choosing to travel by land because of environmental reasons. The possibility to reduce emissions by choosing the least polluting mode of transport. Being able to travel according to one’s own (personal) values
Aesthetics	14	Enjoying the landscapes from the train/bus/car, choosing scenic routes to travel
Spirituality	4	Feeling of making a “right choice” by travelling by land instead of flying

## Conclusions and Discussion

The purpose of the research was to understand the phenomena of travelling by land, and what is the desired consumer value of travelling by land experience by examing a social media group related to the topic. Using Holbrook’s [10] framework of customer value as a base for the analysis, the results of the netnographic study show that travelling by land contributes most to adding self-oriented value for the members in the group. This is demonstrated by the importance of play, efficiency, and excellence value dimensions. Apart from ethics, three other-oriented values (status, esteem, and spirituality) were not often represented. Drawn from this, travelling by land contributes to adding self-oriented value, as the consumption is prized for one’s own sake. Self-oriented means to consider the consumption experience (travelling) for how the person reacts to it, or what kind of effect it has on the person travelling [10]. It does not exclude the consumption experience for providing further types of value involving others as well, but the primary source of value received from, (in this research) travelling by land, comes from its capability to contribute members own consumption experience. However, togetherness [12] further added to complete Holbrook’s framework in this research. Time spent on a train or bus can be used to relax and spend time together with the family or other travel companions. This is also highlighted in the definition of slow travel, as part of travelling to the destination is an essential part of the experience.

The value component togetherness as suggested by Komppula and Gartner [12] was necessary to add for the research, as in many of the posts analysed included referring to travelling together with family and friends, and the time spent together was valued in many of the comments as well. Play value component was the most often identified in the collected data set. This is understandable as travelling is mainly something the members in the group do “for fun” and enjoy in their leisure time, as there was no discussion about the work-related travelling in the data set. Efficiency was also applied to more than half of the collected posts (92). The short travel time is appreciated according to the data collected from the members' discussions in the group. Also, the convenience of travel is important. For example, the changes between trains need to be long enough for there is no rush in running from train to another or risk of missing the next connection. The convenience is also highlighted in the discussion for booking seat tickets in advance, and not to have too long transfers at the time (e.g. sitting in a train/bus for 10 h or more.) The possibility to arrange a route and travel times to fit one’s schedule is important. Concerns about the prices of tickets and the costs of the trips also speak for efficiency being an important value component. According to the posts and comments in the group, it is possible to save money by choosing to travel by land (compared to the flights) but one needs to be ready to compare prices between buses and trains, between different days and times, and between different transport companies as well. One needs to be also in time with the bookings, as the ticket prices increase the closer the travel day becomes. The concerns of time and especially the price are often compared to the flights in the group.

The research findings were surprising, as the pre-assumption when conducting the research was that members value the environmental reasons and chose to travel by land to minimise the emissions and their greenhouse gas footprint. However, the value component “Ethics” was not placed to even to a majority of the collected posts (25 posts out of 185). From this could be drawn that environmental reasons are not the primary reason for travelling by land for most of the members. However, as previously noted in this paper, it can be also since members have already made the conscious choice to avoid flying, which is more consuming for the environment than travelling by land. During this sustainability boom in the tourism industry, it is crucial to understand that the experience itself still stands on top. The consumers choose sustainable options when they create value for the tourists themselves. It is not enough that sustainability is promoted as a way to save the world for others.

As noted by Gallarza and Saura [6] it can be assumed that Holbrook’s typology of consumer value can be utilized to explain a travel experience [6]. This research, however, continues the criticism of the lack of negative value components in Holbrook’s customer value typology [6,12]. Self-oriented dimensions of Holbrook’s typology (Efficiency, Excellence, Play and Aesthetics) of consumer value, are representative of consumer behaviour [6]. This applied in this research as well. Other-oriented values were more difficult to operationalize. As noted earlier in this research as well, the operationalization of esteem and status, and especially separating only one of them to represent the value received from travelling by land was difficult with the current research approach. There were challenges to operationalizing the value dimension of spirituality as well. As noted by Gallarza & Saura [6] it should be studied further in future research.

Since the consumer value typology of Holbrook lacks the negative value components, operationalizing the framework in a service context is challenging. As prior research demonstrates, tourists do not often consider the environment when making transport mode decisions, but instead, they rather focus on minimising the cost and travel time [17]. Yet important aspects of travel mode choice are also comfort, convenience, and flexibility [8]. As noted also by Ram et al. [26] mobility aspects of travelling are often considered a necessary evil of making a vacation trip. This consideration highlights the fact that for some tourists the “real holiday” begins only at the destination, not yet on the way to it [26] Tourists’ concern about time and cost of travel, family commitments and the simple desire to “see the world” can outweigh any consideration of the environmental impact of their travel [9].

This applies also to this research. Those who have decided to travel by land have chosen to avoid flights. However, the reasons behind the decision are not necessarily derived only from environmental concerns or it might be, that motives for joining the group were not discussed in this material. Yet, like the findings in this research show, tourists primarily make the transport mode decisions based on efficiency, considering the time and cost variants of their travel. Yet, for some travellers, the possibility to choose scenic routes and plan the trip according to their own pace was important, yet those were in minority according to the data.

As it was seen in the research finding as well, stories written and published by other travellers can inspire one’s vacation plans and inform the decisions. Tourists may get first-hand knowledge as well as highly relevant information from others who they like and/or perceive as similar to themselves [37]. The members in the Facebook-group can consider the others being similar to themselves, as they have at least one thing in common; the desire to travel by land, avoiding flights. Therefore, they are willing to ask tips from each other in the group and trust each other’s support. According to the data and the posts analyzed, the group Maata pitkin matkustavat is especially important to its members in the pre-trip phase. As Xiang and Gretzel [37] also note, tourists may need to make high-involvement decisions about the products or services, which lack standardization, are difficult to describe, cannot be inspected before purchase and are high in emotional content. For these reasons, experience-based content and information are critical in the context of tourism. Yet, telling about the travels (recollection phase) in the group also enables the members to reflect their experience, and travel narratives can also be an important part of social interactions [37].

There have not yet been many empirical studies in the slow tourism field that would have explored the importance of value for tourists. Earlier studies have constructed frameworks [21] and studied motivations [25] as well as explored travel patterns [19]. This study provides strong evidence on the factors that tourists value in slow travel. Developing possibilities to co-create play and efficiency value with the tourists is paramount for developing slow tourism further. Even though earlier studies have identified motivations important for slow tourism [25], the importance of experienced value has to be also accounted for. The tourists in our study are already motivated for slow travel, but to keep them motivated and enable repeat behavior, slow travel has to be valuable for them. Thus, this study has been able to identify the superordinate goals of slow tourists as called by Oh et al. [25].

It must be noted that since the research only studied traveling by land experience by collecting data from one Facebook- group, the research results can only give directional insights, of the consumer value in traveling by land experience. To complement the research, reasons, and motives behind the travel mode choice of those traveling by land could be studied. Interesting is also the participation in online communities. Also, a different research approach is recommended to find more about the underlying motivations.

## References

[1] Arnould E, Price LL (1993). River magic: extraordinary experience and the extended service encounter. J Consum Res.

[2] Boellstoff T, Nardi B, Pearce C, Taylor TL (2012). Ethnography and Virtual Worlds – A Handbook of Method.

[3] Böhler S, Grischkat S, Haustein S, Hunecke M (2006). Encouraging environmentally sustainable holiday travel. Transp Res Part A Policy Pract.

[4] Dickinson J, Lumsdon L (2010). Slow travel and tourism.

[5] Eriksson P, Kovalainen A (2008). Qualitative methods in business research.

[6] Gallarza M, Saura I (2006). Value dimensions, perceived value, satisfaction and loyalty: an investigation of university students’ travel behavior. Tour Manag.

[7] Heinonen K, Medberg G (2018) Netnography as a tool for understanding customers: implications for service research and practice. J Serv Mark (2018)

[8] Hergesell A (2017). Environmental commitment in holiday transport mode choice. Int J Cult Tour Hosp Res.

[9] Hibbert JF (2013) Understanding the role of the tourists’ identity in travel. Doctoral thesis, Bournemouth University & Linnaeus University

[10] Holbrook M (1999). Consumer value a framework for analysis and research.

[11] Holbrook MB (2005). Customer value and autoethnography: subjective personal introspection and the meanings of a photograph collection. J Bus Res.

[12] Komppula R, Gartner W (2013). Hunting as a travel experience: an auto-ethnographic study of hunting tourism in Finland and the USA. Tour Manag.

[13] Kozinets RV (1997). “I want to believe’’: a netnography of the x-philes' subculture of consumption. Adv Consum Res XXIV.

[14] Kozinets RV (2010). Netnography.

[15] Kozinets RV, Dolbec PY, Earley A (2014). Netnographic analysis: understanding culture through social media data: sage handbook of qualitative data analysis.

[16] Kozinets RV (2015). Netnography: Redefined.

[17] Larsen G (2013). Understanding tourists' perceptions of distance: a key to reducing the environmental impacts of tourism mobility. J Sustain Tour.

[18] LeCompte MD, Schensul JJ (1999). Designing and conducting ethnographic research.

[19] Lin L-P (2017). Industrial tourists' behavioral intention toward slow travel in Taiwan. J Sustain Tour.

[20] Losada N, Mota G (2019). Slow down, your movie is too fast': slow tourism representations in the promotional videos of the Douro region (Northern Portugal). J Destination Mark Manag.

[21] Lumsdon L, McGrath P (2011). Developing a conceptual framework for slow travel: a grounded theory approach. J Sustain Tour.

[22] Maata pitkin matkustavat, Facebook group. https://www.facebook.com/groups/867083580048037/, Accessed 28 Oct 2020

[23] Marcus G (2012) In: Boellstoff T, Nardi B, Pearce C, Taylor TL (eds) Ethnography and Virtual Worlds – A Handbook of Method, pp xiii-xvii. Princeton University Press, Princeton

[24] Mkono M (2013). Using net-based ethnography (netnography) to understand the staging and marketing of “authentic african” dining experiences to tourists at victoria falls. J Hosp Tour Res.

[25] Oh H, Assaf AG, Baloglu S (2016). Motivations and goals of slow tourism. J Travel Res.

[26] Ram Y, Peeters PN (2013). Happiness and limits to sustainable tourism mobility: a new conceptual model. J Sustain Tour.

[27] Sales Oliveira C (2019). My trip in my words: subjectivities, time(s) and mobilities in slow travel blogs. Time Soc.

[28] Sánchez-Fernández R, Iniesta-Bonillo MÁ (2007). The concept of perceived value: a systematic review of the research. Mark Theory.

[29] Sharpley R, Stone P (2012). Contemporary tourist experience : concepts and consequences.

[30] Tavakoli R (2018). Netnography in tourism – beyond Web 2.0. Ann Tour Res.

[31] UNWTO: Tourism’s Carbon Emissions Measured in Landmark Report Launched at COP25. Press release of COP25, 4 December 2019, Madrid, Spain. https://www.unwto.org/news/tourisms-carbon-emissions-measured-in-landmark-report-launched-at-cop25, Accessed 28 Oct 2020

[32] Walls AR, Okumus F, Wang YR, Kwun DJ (2011). An epistemological view of consumer experiences. Int J Hosp Manag.

[33] Whalen EA (2018). Understanding a shifting methodology: a content analysis of the use of netnography in hospitality and tourism research. Int J Contemp Hosp Manag.

[34] Williams P, Soutar G (2000) Dimensions of customer value and the tourism experience: an exploratory study. In O A (ed.), ANZMAC 2000. Visionary Marketing for the 21st Century: Facing the Challenge, Gold Coast, Queensland ed., vol N/A, pp 1415–1421

[35] Visit Finland. Kestävän matkailun linjaus (Strategy for sustainable tourism). https://www.businessfinland.fi/suomalaisille-asiakkaille/palvelut/matkailun-edistaminen/tuotekehitys-ja-teemat/arktinen-kestava-matkailudestinaatio/, Accessed 28 Oct 2020

[36] Wu M, Pearce P (2013). Appraising netnography: towards, insights about new markets in the digital tourist era. Curr Issues in Tour.

[37] Xiang Z, Gretzel U (2010). Role of social media in online travel information search. Tour Manag.

